# Artificial Intelligence as a Strategy in the British Economic Field

**DOI:** 10.1111/1468-4446.13218

**Published:** 2025-04-29

**Authors:** Will Atkinson

**Affiliations:** ^1^ School of Sociology, Politics and International Studies University of Bristol Bristol UK

**Keywords:** artificial intelligence, Bourdieu, domination, field, strategy

## Abstract

Drawing on the sociology of Pierre Bourdieu, this paper conceives the adoption and development of artificial intelligence by businesses as a strategy within the economic field. Using a survey of over 2000 businesses in the UK and tools of geometric data analysis, I construct a model of the British economic field and project into it indicators of past, present and intended AI adoption. This provides a sense of the correspondences between the structure of the field, the temporal order of strategies, and perceptions of the possible and necessary among its agents. Dominant players within the field have clearly led and will lead the AI ‘revolution’, rendering AI a tool for perpetuating intra‐field domination and reproduction, but others below them seem set to pursue emulation strategies to keep up. These conservation strategies may also contain, however, an internal difference between innovation and dependence corresponding with the new and the old within the field.

## 1 Introduction

The various technologies huddled under the label of ‘artificial intelligence’ (AI) have multiplied wildly in the last decade or so and now infuse everyday practice. Consumer preferences, dating decisions, job suitability, state surveillance and more are now apparently curated by manifold algorithms over which no one has clear control and, before long, much of what people do for a living, whether simply to make ends meet and/or to furnish them with a sense of purpose and worth, may be carried out by machines. Unsurprisingly, then, sociologists have begun to pore over the effects of AI for what people experience, think and do and to critically interrogate the causes and consequences of its biases and failures. Fewer, however, appear to have offered a clear sociological account of the *genesis* and *proliferation* of current AI technology and, in particular, of its adoption and development among businesses providing the goods, services and ideas constituting, in large part, the market of symbolic goods in contemporary capitalist social orders. Those that do venture into this territory, often based on theoretical speculation, offer at best only partial accounts.

This paper offers a different way of thinking about the rise and spread of AI: one drawing on the relational sociology of Pierre Bourdieu. His concepts of field, capital, habitus and strategy, I suggest, can help explain not only the effects of AI, as some have already argued, but how current AI technology emerged and the role of economic interests in the process without suffering the problems of other perspectives. Not only that, but we can gain some insight into what drives the recent diffusion of AI via focussed study of the past, present and future take‐up of related technology among businesses. More specifically, I use data from a government‐commissioned survey in the UK to construct a model of what Bourdieu (2005) would describe as the country's economic field and explore existing and anticipated uses of AI understood as indications of so many *strategies* within the field. The model suggests that the so‐called AI ‘revolution’ today may be understood in terms of *conservation* strategies reproducing the structure of dominance in the field. These conservation strategies also contain a modest internal difference between innovation and dependence corresponding with the new and the old.

## 2 Sociological Research on AI and Innovation

AI as a label is generally used to describe a constellation of technologies designed to mimic or replace human cognitive activity, ranging from robots following simple instructions to programmes capable of learning from data—through association, classification and prediction—in order to generate decisions or new information on command. Its rapid spread and advance have sparked fundamental questions about the nature of the human condition and the possible futures of humanity (see e.g. Osborne and Rose 2024), but sociological inquiry, for its part, has largely concerned itself with exploring the saturation of contemporary social practices and power relations with algorithms (see e.g. Beer 2018; Elliot 2019; Kant 2020; Fussey and Roth 2020; Farell and Fourcade 2023; Lindgren 2023). Particularly vexatious has been AI's propensity to reproduce or amplify biases and stereotypes derived from the people or material it learns from (e.g. Forsythe 2001; Noble 2018; Benjamin 2019), but also looming on the horizon is the possibility that AI, like digital technology generally, will become a new tool or resource differentiating people's conditions of existence, their field of possible actions and judgements of worth (cf. Halford and Savage 2010; Fourcade and Healy 2013; Eubanks 2017; Burrell and Fourcade 2021; Fourcade 2021; Savage 2021).

When it comes to explaining the origins and proliferation of contemporary AI technology, however, sociological accounts become thinner on the ground. The basic historical facts are well known—the early growth out of cybernetics and information theory, the symbolic role of the 1956 Dartmouth workshop in establishing AI as a topic of inquiry, the succession of advances and lulls (or ‘winters’) in research, the latest boom in the digital age to handle Big Data and so on—but few have yet offered an explanation of why it might be that these events unfolded as they did and how they might be playing out among different stakeholders. Those that do appear to fall into several camps. First, there are the Marxists, for whom AI is yet another device of capitalist domination aimed at maximising surplus value and/or enforcing ideology. An early example of this line of thought is Berman (1992), but others have updated the argument for Big Data and machine learning (e.g. Impett 2018; Engster and Moore 2020; Burrell and Fourcade 2021; Walton and Nayak 2021; Howcroft and Taylor 2022; Martin 2023; Carchedi 2024), sometimes with a feminist and/or decolonial spin (e.g. Howcroft and Rubery 2019; Hampton 2023). A variant of this angle is the Foucault‐inspired governmentality approach, which imagines AI to be a new technology capable of producing docile subjects in line with neoliberal rationality (Elish and boyd 2018; McKelvey and Roberge 2023). These contributions rightly draw attention to the role of both economic interests and the state, and some sensibly suggest differences between sectors and company sizes in the applications and expansions of AI, but most are based on theoretical assumption or assertion more than anything else, or at best case studies of companies or industries and general discussions. They also tend to be reductive in explaining the varied interests in developing and employing AI when putting it all down to exploitation or ideology, especially among scientists and state actors but even among business owners, many or most of who will be driven by sincere belief in their product/service or competitive rivalries rather than a quest to dominate or mollify workers. By contrast, science and technology studies (STS) practitioners, admittedly of varying conceptual persuasions, often document the distinctive values, interrelations, conflicts and (gender) inequalities among AI researchers but are generally more concerned with the constitution of AI as a ‘thing’ and its place within networks of activity, or the flawed or biased assumptions of researchers, than how and why the processes associated with this ‘thing’ are taken up and used by varied others in line with their specific interests and ideas (e.g. Fleck 1987; Forsythe 2001; Collins 2018, 2021; Suchmann 2023; Wajcman and Young 2023).

Broader currents in economic and organisational sociology, if not addressing it directly as yet, might offer some clues on how else the rise of AI might be explained. Of those interested in explaining the origins and take‐up of ‘innovations’—organisational as well as technological—the neo‐institutionalist approach advocated by DiMaggio and Powell (1983; Powell and DiMaggio 1991) stands out, not least because of Powell's related research on the biotechnology sector (e.g. Powell 1996, 1998, 1999; Powell et al. 1996). The basic drift of the approach is that organisations are situated within so many ‘fields’ of activity—some characterised by open and direct competition and others implicating regulatory bodies, research institutions and pressure groups—generating tendencies toward homogenisation (or ‘isomorphism’). Development and early adoption of novel processes and technologies may be driven in part by competition and differentiated by firms' resources and competencies—in his empirical work, Powell puts particular emphasis on the capacities to forge and maintain interorganisational connections and interdependencies and to process what flows through them^1^—but subsequent diffusion of those innovations through other organisations in weaker or dependent positions in a regulated field rests instead on coercion from or mimicry of field leaders and regulators as well as emerging norms. Although specific conditions may foster ‘institutional entrepreneurs’ in this context (Greenwood and Suddaby 2006), fields thus tend toward conformity and inertia, even—or especially—where technology is developing rapidly. A similar conceptual architecture, with some further distinctions and nuances, has been elaborated by Fligstein (2018; Fligstein and McAdam 2012), who likewise emphasises that competitive markets tend toward stability because of a human impulse for control and certainty.

Neo‐institutionalism is certainly less reductive than Marxism and less descriptive than STS, but it tends toward conceptual and explicatory vagueness (Alvesson and Spicer 2018). Its conception of field‐specific resources and capacities—including the profits to be gained from interorganisational ties—is underspecified and the precise structure of its posited fields, beyond asymmetric interdependencies, is unclear. The locus of investigation is typically a specific sector or industry rather than the wider diffusion of an innovation across sectors, moreover, and its evidence base consists largely of historical or contemporary case studies like Powell's. These can capture important details of organisational operations and interactions, for sure, but they struggle to grasp with any precision the broader, *latent* system of relations—hierarchies and oppositions—within and across sectors in which interactions are embedded. The variety of organisations implicated in a regulated or ‘institutionalised’ field also seems too diverse, encompassing actors—entrepreneurs, regulators, scientists, etc.—likely to have very different interests, desires and frames of reference.

An alternative perspective, joining up, filling out and reframing the others, is offered by the relational sociology of Pierre Bourdieu. For this offers not only a means of capturing the myriad effects of AI on practices and inequalities, as some have already begun to suggest, but an integrative mode of explaining the very birth and spread of current AI technology. It can illuminate the web of relations between science, the state and business involved without reducing the interests and practices of one to another while enabling us to rigorously model the desires, hierarchies and struggles between businesses as a system with some—not total—autonomy from class relations. In short, developing and adopting AI technology becomes a strategy not simply for exploiting or pacifying workers, nor for maintaining stability and certainty, but for winning or preserving recognition in the competition for the specific stakes of the business game.

## 3 Outline of a Field‐Theoretical Account of AI

Bourdieu's (2000) starting point is the human quest for diversion and purpose, especially in the form of recognition as worthy beings from others. Since worth is often associated with distinction (in the sense of difference), historically this has resulted in struggles to impose specific properties and possessions—and their possessors—as inherently worthy of recognition as against others. Bourdieu labelled these properties and possessions ‘capitals’ and, in contemporary societies, they lie at the heart of several autonomous domains of practice that Bourdieu—predating neo‐institutionalism—called ‘fields’. Each field has its own capitals, its own hierarchies structured by their possession, its own taken‐for‐granted rules of the game in the struggle for capitals (doxa), its own draw on people's desire and commitment (illusio) and its own effects on people’ sense of the possible and desirable in relation to the struggle (habitus), which in turn generates different species of activity—subversive or conservative, depending on position—for attaining capital, and recognition, in the field (strategy).

One such field is the national class structure, or social space, structured by possession of economic capital (money, financial assets) and cultural capital (mastery of legitimatised sign‐symbol systems like art, languages etc), the latter also containing a variant ‘technical’ capital premised on certified mastery of techniques and technical systems (Bourdieu 1984, 2005). Habitus in the social space translates into a sense of the possible and desirable in relation to jobs, savings/investments or education but also moral‐political ethos, tastes and associations of specific phenomena (practices, goods, etc) with specific classifications and evaluations (vulgar, distinctive, etc). AI is frequently trained on these schemas, as well as those associated with ethnoracial and gender categorisation, and in turn consolidates, amplifies and embellishes them (see e.g. Airoldi 2022; Hristova 2023).

To explain the *emergence* and *proliferation* of today's AI technology, however, we need to go beyond class, race and gender to recognise the existence of multiple other fields with relative autonomy. Each revolves around one or more distinct form of capital—form of esteem and value, that is—specific to the field but with outside or ‘heteronomous’ capitals like money still playing their part. Among these fields are the field of politics (Bourdieu 1991), the state as a ‘bureaucratic’ field (Bourdieu 2005, 2014), the scientific field (Bourdieu 1975/1981, 2004) and the field of businesses, or the ‘economic field’ (Bourdieu 1996, 2005), all of which (along with other fields) sit within an overarching ‘field of power’ wherein the major forms of legitimacy and value are struggled over (Bourdieu 1996). The state occupies a central place within this field insofar as it has ultimate authority over the value and regulation of capital in all fields, including the economic field, which is exercised through legitimation circuits, that is, networks of actors delegated to represent the central authority (Bourdieu 1996, 2014). Members of different fields may form cross‐field alliances and networks of interaction and exchange depending on their interests and their necessities. In some cases these networks may revolve around specific domains or tasks—housing policy is an example from Bourdieu's (2005) own work—in which case it can be useful to describe them, after Becker (2006), as ‘worlds’.^2^ This captures with greater precision what neo‐institutionalists seem to have in mind when describing institutionalised fields implicating interdependencies between multiple types of organisations.

If the scientific field is oriented around peer esteem in the quest to establish truth—and, for some, to develop useful technologies on that basis—and the political/bureaucratic fields are structured around conceptions of political/administrative competence, the economic field is largely oriented toward the logic of profit but also, to different extents for different agents, implicates earnest belief in the importance of the product or service one intends to supply (i.e. illusio). The agents or players in the field are businesses, many of which, particularly larger ones, comprise micro‐fields of their own in which delegated actors act or think ‘on behalf of’ the business in the economic field (Bourdieu 2005, 205–7). Bourdieu (2005, 194–5) suggested a number of capitals these actors may use in their struggle to maintain or improve their position in the economic field. The most important of these is *financial* capital (earnings and fiduciary assets), but he also mentioned *technological/technical* capital (materials and people implicated in the design and manufacture of products), *organisational/commercial* capital (mastery of information, marketing and infrastructure), *social* capital (connections and interdependencies with other firms, suppliers etc.) and *symbolic* capital (brand name, lineage, company listing, ‘honour’ etc).^3^ Elsewhere Bourdieu (1999, 126–7) also talked about the ‘profits of localisation’, or the advantages accruing to occupation of or access to a specific geographical site.

Today the economic field is global in scale, but national economic fields, regulated by a state, nonetheless constitute subfields of it and retain some autonomy insofar as not all local players are recognised on the global level and, indeed, the local/global opposition in a national field becomes a meaningful principle of organisation (Bourdieu 2005, 223–32). Moreover, both the global and national economic fields are structured into a plethora of subfields roughly coterminous with what might be thought of as different ‘sectors’ or ‘industries’, each with their own specific histories, variants on the illusio/capitals of the parent field and thus meanings attached to such things as prices or technologies, though these subfields may merge or break apart depending on the state of struggle and interdependencies (Bourdieu 2005, 194, 203–4). At the same time, an economic field and its subfields, like all fields, are structured according to the established/challenger or old/new opposition (Bourdieu 2005, 201), which often maps on to conservative/subversive strategies, but many so‐called ‘revolutions’ in the economic field—innovations, market transformations etc.—are typically led by larger firms able to pair technological capital with other forms of capital operative within the field in order to maintain their position (Bourdieu 2005, 203).

The history of contemporary AI technology, both its genesis and diffusion, could surely be rewritten in terms of struggles and strategies within and between several national and international fields constituting a specific AI world, mostly notably, since these are the major stakeholders of AI identified by others, the scientific field, the political‐bureaucratic fields and the economic field (Hoffman 2017; Eynon and Young 2021).^4^ Its origins among cyberneticists and information/computation theorists, for example, indicate its birth in (a subfield of) the transnational scientific field, in line with the stakes and positions there, and the very constitution of AI as a distinct scientific entity, or class of associated materials, processes and symbols subject to systematic inquiry, was anchored in a strategy among its advocates to position themselves against automata theory, cybernetics and the dominant figure of Norbert Wiener (Nilsson 2010). Both Bloomfield (1987) and Fleck (1987) then document the subsequent ‘thought styles’, divisions and competition between AI researchers and labs which can be reconstructed as dispositions, struggles and strategies within the scientific field. Almost from the start, however, the development of AI implicated the economic field: the Dartmouth workshop, usually considered the founding event of AI as a realisable thing, was co‐organised by Nathan Rochester of IBM, then as now a company specialising in providing information processing systems to other businesses and therefore reliant on developing technological capital to manage its place within the economic field. Later advances and ‘winters’ in AI research then depended largely on the extent to which states and businesses invested in the technology (Fleck 1987; McCorduck 2004). States did so—the Defence Advanced Research Projects Agency (DARPA) in the US was an early investor, for example, and later the British and Japanese states were heavy funders—because the technology promised to improve defence, surveillance and economic performance, as strategies oriented at once toward maintaining a state's control and accumulation of all capitals within its territory (Bourdieu 2014) and preserving or improving its position within the field of nation states (Schmitz et al. 2023), but funding abated when progress was considered too slow.

Businesses, for their part, will have invested in AI technology insofar as it promised to improve or maintain, in one way or another, their capital in the economic field. The finance sector, booming in the West from the 1980s onwards, was an early investor, specifically in ‘expert systems’, as a means to reap financial capital from mastery of markets (Fincham et al. 1994; McKenzie 2021). Now in the computer and digital age, with the rise of companies such as Google, Amazon, PayPal, Facebook and Netflix (each with their own sociological histories to be told), this promise has increasingly taken the form of handling and processing Big Data, through machine learning, as a means of improving organisational/commercial capital through, for example, generating knowledge on customers and tailoring recommendations (see Srnicek 2017). As investment in AI has intensified, so those firms specialising in developing it—Nvidia being the most prominent/dominant among them—have profited and surely risen in the global economic field. At the same time, new potential applications of AI technology to the arts and education have surfaced, such as AI‐generated scripts, essays, papers, poems or artworks. These have become points of observable differentiation, in the form of (qualified) embrace or rejection, within the intellectual field—by artists and academics, for example—but they also offer new spaces for enterprise within the economic field (see Patel 2020; Lee 2022, 2024).

Herein lies the point: the meaning and use of AI among businesses will vary according to their position and trajectory in the economic field. Investment in AI can be understood as a part of a *strategy* to accumulate or maintain capital, but what and how much one invests, what types of AI are invested in and whether one is a ‘leader’ of a ‘follower’ are likely to be adjusted to the volume and structure of capital possessed as well as the specific subfield occupied. These are not understood to be simply conscious calculations of how best to maximise surplus value or pacify the masses, though some may conceivably operate with that mindset (as itself a socially produced scheme of perception), but practices flowing from the perceptions of the possible and desirable in the struggle for the specific form of misrecognition—purpose and worth—offered by the economic field: being a successful business, offering goods and services perceived as valuable, and besting or keeping up with competitors. And while AI might well reproduce and embellish dominant categories of thought and lead to job losses and insecurity for many, it may also operate as a principle of domination, and reproduction of dominance, *within* the economic field.

These propositions—that business orientations toward AI will differ systematically according to position and trajectory in the economic field and may reproduce intra‐field domination—are not merely assumptions or assertions but parts of a rational construction of the object open to empirical confirmation or revision (Bourdieu et al. 1991). We can, in principle, go beyond common accounts relying on piecemeal evidence or guesswork and actually test whether and how business strategies are differentiated by embedding them within an appropriate model of the economic field. Fortunately, data exist which allow us to do precisely that in the British context. It does not illuminate the longer history of AI or disclose directly the wider workings of the AI world, the broader dynamics of the global economic field or what has been going on in dominant states, that is, the US and China, but it does enable us to investigate with some rigour and detail a specific part of the overall picture: the manner in which businesses have contributed to the recent proliferation of AI, and the possible consequences for the economic field of their doing so, within a nation state not only relatively prominent in the field of nation states (Schmitz et al. 2023; Atkinson 2022), suggesting notable weight in the global economic field, but widely regarded as the third force in contemporary AI investment and innovation.^5^

## 4 Data and Method

The data derive from a survey commissioned by the UK Department of Digital, Culture, Media and Sport (DCMS) Office for Artificial Intelligence and delivered by YouGov in 2021 (*n* = 2009).^6^ Both the Office and the survey, the primary aim of which was to model potential for AI growth in the economy, are themselves indicative of the British state's tactics for managing the national economic field and its capital in the field of nation states. Being carried out in 2021, the survey coincides with the very start of the current ‘AI boom’ or ‘spring’ and offers an interesting snapshot of the conditions of possibility for what has come after.

Information on sampling and recruitment is regrettably sparse but the survey was a web‐based self‐completion questionnaire covering businesses in England, Wales and Scotland—Northern Ireland has been excluded. The unit of analysis, as for Bourdieu (2005, 2008), is the business rather than specific human individuals. While this does mean intra‐organisational struggles, which would reveal much about particular strategies, must be bracketed, it is appropriate since businesses are, in the economic field, the assemblages to which relevant capitals are attached and human perceptions—and collective strategies—attuned. Only private sector enterprises were sampled, meaning state‐owned enterprises are missing, but these are scarce in the UK context anyway.

The first step is to construct the model of the economic field using specific multiple correspondence analysis (MCA), the technique adopted by Bourdieu as a natural counterpart to his topological conception of fields and now fairly well established in European sociology.^7^ MCA operates to identify patterns of association and opposition between categories (modalities) of nominal variables—what tends to go with what and what does not—and array those patterns spatially, in terms of proximity/distance, with different axes of association/opposition accounting for differing proportions of the overall variance in the data (Le Roux and Rouanet 2004, 2010; Hjellbrekke 2019). In our case, four variables yield 20 active modalities for the construction of the space (see Supporting Information S1: Table S1). These are (i) estimated annual turnover, as a rough indicator of *financial* capital; (ii) FTSE listing, as not only another indicator of financial capital but the *symbolic* capital of inclusion in an exclusive classification; (iii) region of main operation, as a measure of relative access to advantageous infrastructure, connections and markets (especially in terms of distance from the centre of capital, London), which might be taken to indicate specific forms of *commercial/organisational* capital as well as *profits of localisation*; and (iv) industry of operation, as a means of factoring in the arrangement of *subfields* within the economic field (Table 1).^8^ Company size, as measured by number of employees, is included as a supplementary variable.

**TABLE 1:** Active variables in the MCA model of the British economic field.

	*n*		*n*
Turnover		Industry	
< £1m	715	Retail	226
£1–9.9m	330	Info and Comms Tech (ICT)	270
£10m+	624	Construction	198
Missing	340^a^	Arts and leisure	143
		Finance	270
Region		Manufacturing, mining, agriculture	254
East	131	Infrastructure	85^a^
London	406	Education, health, social services	267
Midlands	265	Other	192
North	445	Missing	104^a^
Scotland	151		
South	518	Size (employees)	
Wales	76^a^	Micro (< 10)	775^a^
Outside UK	17^a^	Small (10–49)	342^a^
		Medium (50–249)	291^a^
FTSE listing		Large (250+)	601^a^
Not FTSE	1721		
FTSE 250	75		
FTSE 100	91		
Missing	122^a^		

^a^ Categories are set as passive in the MCA.

Further or more specific information on forms of capital is absent, meaning some differences and deviations in the field are likely to be hidden by the model. This is a hazard of relying on secondary data. However, since the variables available do measure basic forms of what might be considered primary capitals in the field, as well as sectorial segmentation, we can be confident that the model offers a sufficient approximation of core principles of difference within the field. The omissions do not invalidate the model, therefore, but only make it a less precise approximation of field structures than could otherwise be the case: broadbrush rather than refined, and slightly distorted, but an approximation nonetheless.

The second step is to project into the space, as supplementary categories, responses to various questions about the use of AI. These include past, present and anticipated uses of five types of AI: machine learning; natural language processing and generation (e.g. chatbots, translation); vision/image processing and generation (e.g. facial recognition, image generation); data processing (e.g. analysis, forecasting); hardware (e.g. autonomous machines, drones); and robotics (e.g. non‐learning automation). There is also a variable indicating whether businesses have developed AI technology in‐house, outsourced development or bought readymade software, which is taken as an indicator of different tactics adjusted to the firm's capital profile, especially its *technological* capital in relation to economic and social capital. Variables related to past expenditure on AI—on both technology and labour—give further information on the specific investment of capital, and variables on projected future expenditure reveal the sense of the game and expected unfolding of strategies. Distances between and coordinates of categories in the space are assessed using standard tools of geometric data analysis (GDA): test‐values, scaled deviations (SDs) and t‐tests (Le Roux and Rouanet 2004, 2010; Lebart et al. 2006; Hjellbrekke 2019; Le Roux et al. 2020).

Most businesses in the UK, at the time of the survey, were not using AI technology and had no plans to (Table 2), though that might have changed in the meantime. Between 20% and 30% used AI in some form and smaller proportions were either piloting AI or planning to use it in the future. Data processing and analysis is the most widely used form of AI and hardware is the least widely used. Of those already using AI, around half have spent less than £100,000 on it over the last 3 years and only about a fifth have spent more than £1 million (Table 3). Substantial proportions of them expected to increase their expenditure on AI by up to 50% but some anticipated doubling their expenditure of even quintupling it (Table 4).

**TABLE 2:** Business uptake of forms of AI (%).

	Machine learning	Language processing	Image processing	Data analysis	Hardware	Robotics
Not used, no plans to	63.8	64.1	71.4	54.8	73.0	65.2
Not used, planning to	9.5	9.5	6.4	11.5	7.0	7.0
Piloting	4.9	5.9	4.4	5.3	4.7	4.0
Have used < 3 years	12.7	11.7	10.2	14.6	7.8	11.5
Have used 3+ years	9.1	8.8	7.5	13.8	7.4	12.2
Total	100.0	100.0	100.0	100.0	100.0	100.0
*n*	1753	1754	1757	1745	1759	1775

*Note:* Missing data excluded.

**TABLE 3:** Costs of AI technology and labour in the last 3 years (%).

	Technology	Labour
< £10k	26.7	24.8
£10–49k	13.4	12.0
£50–99k	14.4	13.8
£100–499k	15.0	15.9
£500–999k	9.6	13.0
£1–9.9m	12.9	12.8
£10–19m	4.8	4.8
£20m+	3.3	2.9
Total	100.0	100.0
*n*	521	484

*Note:* Missing data excluded.

**TABLE 4:** Anticipated increase in rate of expenditure on AI (%).

	Next year	Next 5 years
No increase	12.0	9.6
Increase from 0	9.5	5.7
< 10%	30.5	10.6
10%–24%	22.0	25.5
25%–50%	9.5	16.3
51%–75%	5.6	11.5
76%–100%	4.8	7.8
101%–200%	2.8	6.2
200%–500%	1.2	3.5
> 500%	1.9	3.2
Total	100.0	100.0
*n*	567	564

*Note:* Missing data excluded.

## 5 The Structure of the British Economic Field

**FIGURE 1:**
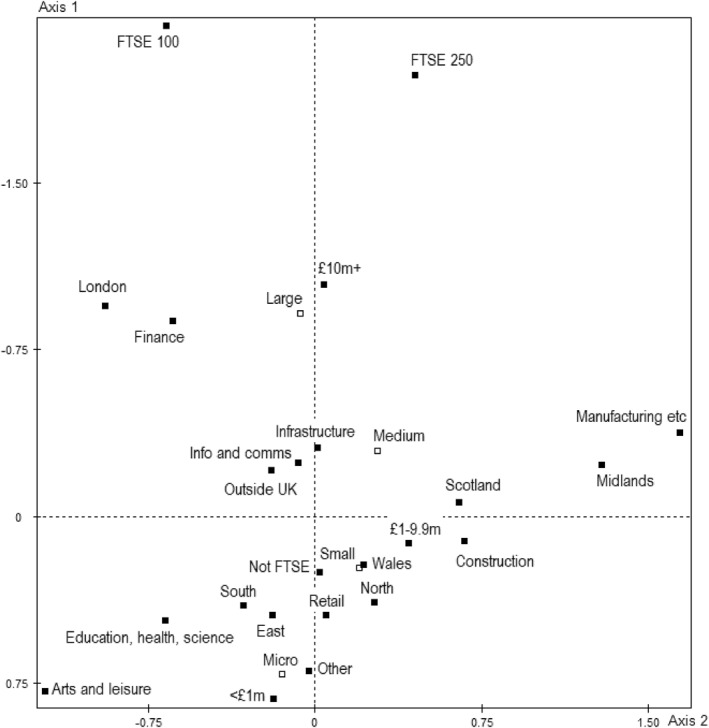
MCA model of the British economic field.

The MCA model of the economic field reveals a space structured in two dimensions with a cumulative modified inertia rate of 0.94 (Table 5). The first axis accounts for the vast majority of this and can be interpreted as an axis of capital volume, particularly volume of financial capital. It thus opposes small companies with low annual turnover and large companies with high annual turnover and, especially, those that are FTSE listed (Figure 1, Table 6). Coupled with this opposition are polarisations of regional connectivity and sectorial subfields. Being situated in London, with its geographical concentration of capitals, is associated with high capital whereas most non‐London regions are associated with lower capital. Finance is situated toward the top of the space and bears an above‐average contribution to the axis, signifying its dominant position in the contemporary British economic field. Sharply opposed to it are businesses operating in the subfields of arts and leisure; education, health/social services and science; and retail (though they do not contribute substantially to the axis inertia, the test‐values for the latter categories nonetheless indicate a powerful association with the low‐capital pole). These are the dominated subfields of the economic field. Construction, infrastructure (energy, water, etc) and information/communications technology (ICT) companies occupy intermediate positions on average, though the latter two are, according to test values, associated with the dominant pole overall. Their middling locations reflect the pronounced dispersion of ICT/infrastructure firms along the axis (see Supporting Information S1: Figure S1).

**TABLE 5:** Axis inertia.

Axis	Eigenvalue	Inertia %	Modified eigenvalue	Modified inertia rate	Cumulative modified inertia
1	0.3869	9.5	0.0188	0.87	0.87
2	0.2884	7.0	0.0015	0.07	0.94
3	0.2752	6.7	0.0006	0.03	0.97
4	0.2685	6.6	0.0003	0.02	0.99

*Note:* Benzécri‐modified eigenvalues and inertia rates.

**TABLE 6:** Contributions and test values of modalities in the MCA.

	Contribution		Test value	
	Axis 1	Axis 2	Axis 1	Axis 2
Turnover				
< £1m	**15.63**	1.05	27.46	−6.13
£1–9.9 m	0.16	2.53	2.41	8.38
£10m+	**21.69**	0.04	−31.27	1.19
Missing	—	—	1.14	−1.91
Region				
East	0.83	0.20	5.26	−2.22
London	**11.60**	**15.52**	−21.26	−21.23
Midlands	0.44	**19.05**	−3.97	22.55
North	2.16	1.40	9.28	6.45
Scotland	0.02	2.74	−0.80	8.28
South	2.69	2.31	10.62	−8.50
Wales	—	—	1.95	1.96
Outside UK	—	—	−0.86	−0.80
FTSE listing				
Not FTSE	3.51	0.04	27.59	2.59
FTSE 250	**9.47**	0.66	−17.49	3.99
FTSE 100	**14.22**	1.76	−21.52	−6.54
Missing	—	—	−7.87	−1.28
Industry				
Retail	1.44	0.03	7.11	0.83
Info and comms	0.49	0.06	−4.21	−1.30
Construction	0.08	3.88	1.67	9.99
Arts and leisure	2.86	**9.10**	9.78	−15.07
Finance	**6.66**	4.75	−15.47	−11.28
Manufacturing, mining, agriculture	1.14	**29.64**	−6.38	28.04
Other	2.99	0.01	10.13	−0.37
Infrastructure	—	—	−2.90	0.13
Education, health, social services	1.90	**5.21**	8.24	−11.80
Missing	—	—	−7.31	−1.38
Size (employees)				
Micro (< 10)	—	—	25.28	−5.17
Small (10–49)	—	—	4.72	4.09
Medium (50–249)	—	—	−5.35	5.25
Large (250+)	—	—	−26.64	−1.90

*Note:* Above average contributions are in bold. Test values greater than ±2 are notable.

The second axis captures further regional and sectorial oppositions.^9^ Manufacturing (and secondarily construction) stands opposed to finance, arts/leisure and education/health/social/scientific services and, corresponding with this polarisation, Midlands‐based companies (and secondarily Scottish and Northern businesses) stand opposed to London. This suggests a secondary manifestation of the centre/province polarity, and differences in organisational/social capital and profits of localisation, but given the starkly contrasting pasts of the implicated industries over the last few decades of deindustrialisation—the decline of manufacturing/mining/agriculture and its geographical hubs and the rise of (London‐based) services and, especially, finance—it might also be said to represent opposing *trajectories*. That the opposition coincides with a split between FTSE 100 and FTSE 250 companies at the top (the SD is > 1.0) suggests this is indeed a secondary axis of domination.

**FIGURE 2:**
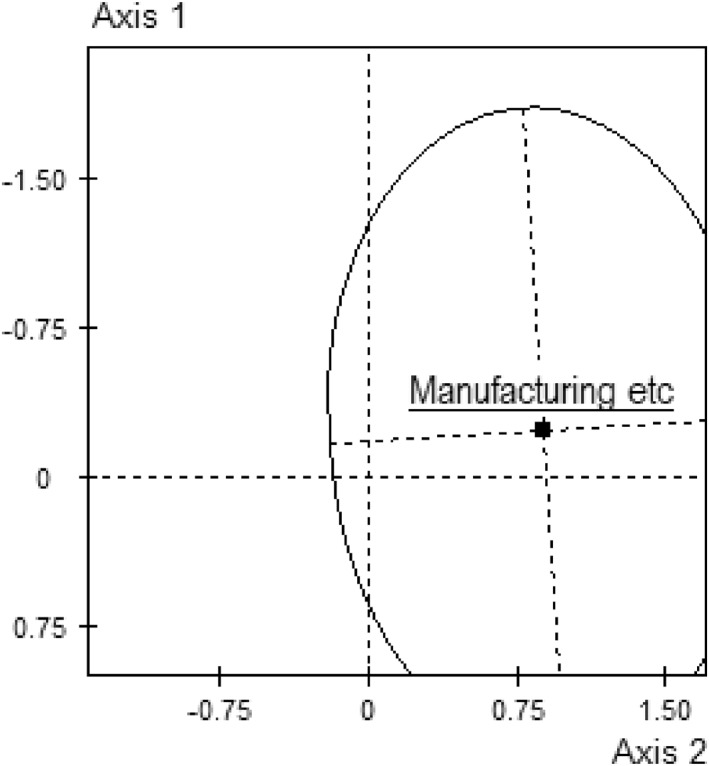
Concentration ellipse for manufacturing in the space. A concentration ellipse gives graphical representation to ±2 standard deviations on the axes.

The plane as a whole represents a tripartite structure. Manufacturing companies, almost exclusively, occupy the entirety of the provincial/declining pole of the second axis, internally differentiated according to their volume of capital (Figure 2), while finance—geared toward the competitive management of economic capital—occupies the top left quadrant and arts/leisure and education/health etc—more closely associated with cultural capital—occupy the bottom left. It is tempting to think of this, as Bourdieu might, as a contemporary manifestation within the British economic field of the ubiquitous Dumezilian triad of *laboratores*, *bellatores* and *oratores*.

## 6 AI as Strategy: Reproduction and Emulation, Innovation and Dependence

**FIGURE 3:**
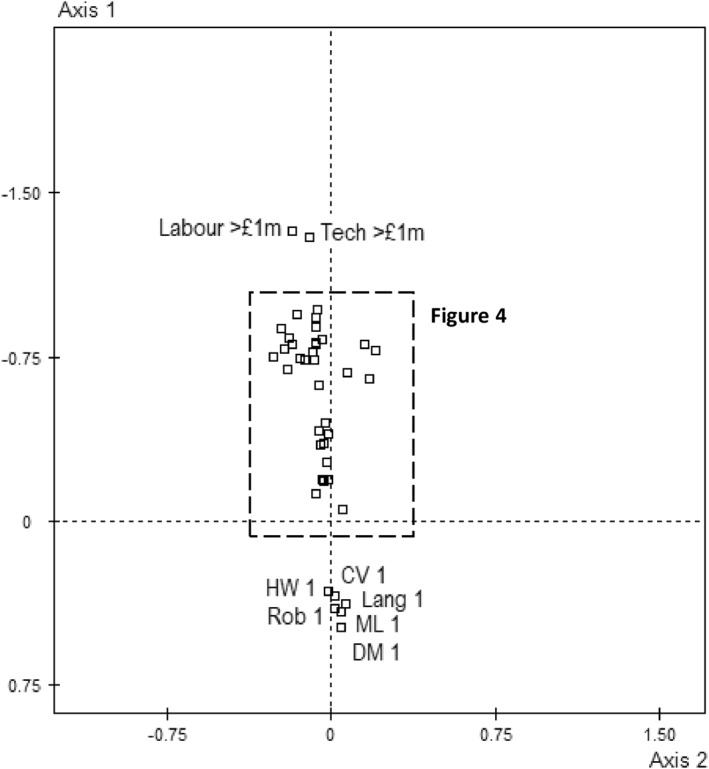
Orientations toward AI in the space. Supplementary variables. 1 = do not use and no plans to, CV = computer visualization, DM = data management, HW = hardware, Lang = language processing, ML = machine learning, Rob = robotics.

**FIGURE 4:**
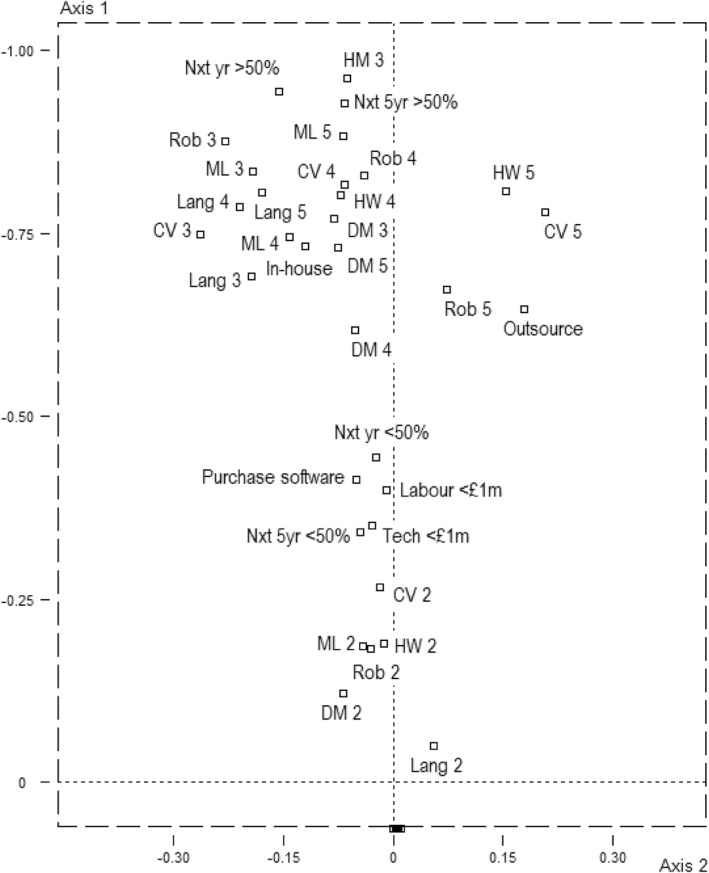
Orientations toward AI in the space (magnified). Supplementary variables. 1 = do not use and no plans to, 2 = not currently using but plan to, 3 = piloting, 4 = used < 3 years, 5 = used > 3 years, CV = computer visualization, DM = data management, HW = hardware, Lang = language processing, ML = machine learning, Rob = robotics.

Past, present and anticipated future uses of AI, indicating phases of strategies within the field, clearly correspond with volume of capital (Figures 3 and 4; for test values, see Supporting Information S1: Table S1). Small businesses with few resources are unlikely to have adopted or to have plans to adopt any form of AI. In contrast, early adopters or those currently piloting AI technology are closely associated with the dominant pole of the first axis populated by larger, higher‐turnover companies typically situated in London. There is further vertical differentiation among AI adopters/developers in terms of how much capital they have invested in the strategy: the more prosperous the business, the more financial capital spent on associated technology and labour power (distances between those spending under and over one million pounds are almost large, i.e. 1.0 SDs).^10^ Likewise, those with ample financial resources are likely to increase their spend on AI by over 50% in the coming years (sometimes over 100 or even 500%) while those with fewer resources are more likely to increase their spend by less than 50% (which includes raising costs above zero).^11^

What all this suggests is that the AI ‘revolution’, as an investment of economic capital in technological capital aimed at reaping greater financial capital, is, in fact, a conservative strategy insofar as it reproduces the structure of dominance within the field. The dominant, their perception of possibilities informed by their capital stocks, can afford to invest in AI to entrench their dominance while the dominated are and know themselves to be unable to. Meanwhile, those businesses that have not yet invested in AI but are *planning* to are associated with the middle region of the space. This perception of the forthcoming suggests an adaptation to the changing state of the game wrought by the rapid development and adoption of AI among dominant players; a shifting sense of the necessary and desirable induced by field effects among those with sufficient capital to render AI adoption objectively and subjectively possible. These are agents who, observing the dominant and seeking to ‘keep up’ and ‘get on’ while ‘getting ahead’ of others, intend to pursue *emulation* strategies—a species of conservation strategy, ultimately, that reproduces the structure of the field by recognising the practices and strategies of the dominant as desirable. If this is the ‘mimicry’ or ‘conformity’ suggested by neo‐institutionalists, now we see its specific social location: among ‘petit‐bourgeois’ businesses displaying a form of goodwill.

**FIGURE 5:**
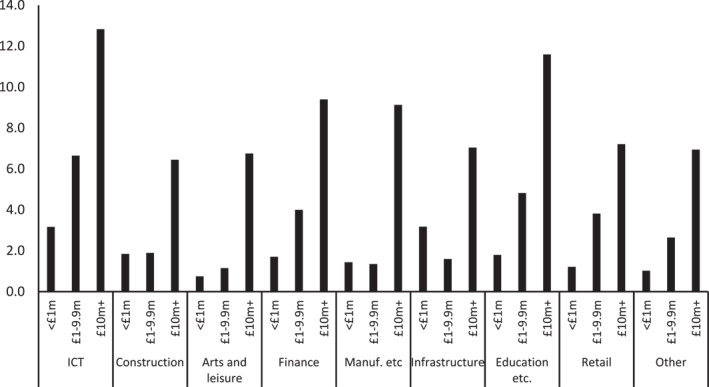
Average AI adoption scale scores by sector and turnover.

Although sectors, or subfields, are polarised along the first axis of the space, it bears emphasising that they are also *internally* dispersed between the poles. Some finance related enterprises—around a third, in fact—have an annual turnover of less than £1 million, for example, and 17% of arts and leisure‐based businesses have a turnover of over £10 million. These differences translate into intra‐sectorial differences in the pursuit of AI technology. To demonstrate this parsimoniously, the individual questions on forms of AI adopted have been aggregated to construct an additive scale representing temporal orientations toward the technology as a whole, ranging from no plans for any form of AI through plans for or piloting various forms of AI to the limit point of having used all forms of AI for over 3 years (range 0–24, mean 4.8, st.dv. 7.2, Cronbach's alpha 0.94). A simple comparison of average scores broken down by sector and turnover shows that those with high revenue in all sectors are more likely than direct competitors in their subfields to have invested or be in the process of investing in AI (Figure 5). For sure, there are still differences between sectors among high‐turnover firms—compare, for example, ICT against construction or arts and leisure—and even medium‐revenue ICT firms are investing at similar speeds or rates to high‐turnover firms in other sectors. Still, turnover per se rather than sector seems to be the biggest factor differentiating investment. Perhaps surprising is the intra‐sector difference for education, health, social services and science (which, no doubt, combines several subfields): firms there turning over £10 million or more per annum (21% of the category) are second only to similarly capital‐rich ICT firms in their speeds and rates of investment. Some sectors are more internally polarised than others in their orientation toward AI, then, modulating the *meaning* of AI as a strategy—as necessity or luxury, for example, or as more or less common among competitors—within any specific subfield.

A further mode of differentiation in the uptake and meaning of AI, returning to Figure 4, is present, albeit modestly, on the second axis of the space. Among those already using or piloting AI, there is some horizontal separation between types of AI adopted and stages of development. Not all test values are significant or deviations notable, but some are and it comes down to an opposition between, on the one hand, the long‐time adopters of hardware and imaging technology (and to a lesser extent robotics), associated with the declining manufacturing pole of the space, and, on the other hand, early adopters of language‐modelling technology and those currently experimenting with various forms of AI, which is associated with the London/finance/arts/education etc. pole of the space and may represent more recent and ‘innovative’ developments and applications of AI. It seems as if this is capturing subfield differences in *older* and *newer* uses of AI mapping onto trajectories—another dimension to the leader/follower opposition.

This horizontal differentiation dovetails with a modest difference in the *mode of investing* in AI. Those AI‐adoptive companies associated with the new/innovative pole of the space are slightly more likely to develop the technology in‐house, indicative of a certain stock of technological and technical capital—that is, materials and people capable of developing technology—being invested in its own augmentation. In contrast, outsourcing the development and maintenance of AI technology to another company—relying on economic capital and social capital—is associated with the old manufacturing pole and signals the increased *dependence* of the sector on others, specifically the ICT (and possibly finance) sectors, and thus differences in power.^12^ Returning to the premier axis, however, both in‐house development and outsourcing are positioned above the other option of buying readymade software ‘off the shelf’ and hoping for the best.^13^ Closer to the middle region of the space, and perhaps signifying a relative lack of technological/technical, social and economic capital, it might be thought of as a sort of petit‐bourgeois emulation strategy akin to the off‐the‐peg rather than tailor made suit or buying champagne from the supermarket rather than sourcing from a specialist merchant or, indeed, from one's own vineyard (cf. Bourdieu 1984).

## 7 Conclusions

AI suffuses everyday life, building on, consolidating and amplifying classed tastes, dominant schemes of perception and unequal distributions of capital. That much has been well established. A joined‐up explanatory sociological account of its rise and proliferation, however, has been less forthcoming. I have suggested that Bourdieu's sociology might offer a solution—confirming the aptness of his conceptual tools for the digital age, as others have done before (e.g. Ignatow and Robinson 2017; Cveticanin et al. 2024)—and sketched some basic outlines of what that might look like. It comes down to unravelling the structures, struggles and strategies involved in the AI world, particularly those of the scientific field, the political‐bureaucratic fields and the economic field. Such an account, avoiding the reductions, omissions and imprecisions of alternatives, could and should be filled out via thorough empirical and historical analysis of these fields and their interconnections both at the global level and within dominant states. Our own focus, however, pivoted to a specific piece of the puzzle, using an opportune government survey in the UK to document recent uses of and plans for AI in the British economic field. Limited as the data is, it yielded insight into what has driven and differentiated the propagation of AI since the start of its latest boom period in one of the most pro‐AI environments in the world.

It might be inferred that, deriving from 2021, the data correctly anticipated the growth and diversification of AI since then. Leading the way have been the dominant players within the economic field, and within each subfield thereof, who therefore reproduce their dominance insofar as they are the ones most able to innovate by investing financial and technological capital.^14^ Other ‘petit‐bourgeois’ businesses expected to follow suit, as much as their resources allowed, in order to keep up with the dominant. Yet there was not blanket conformity or mimicry across the field. There were also many dominated players within the field, disproportionately clustered in specific industries, who, at the time of the survey, did not think it possible or desirable to invest in AI.

As much as AI may transfigure aspects of everyday life, therefore, and eventually transform the nature and availability of employment across fields, it is also a conservative force, maintaining the broad structures of the economic field. Touted as a ‘disruptive’ technology it may be, but disruptive of extant hierarchies and inequalities it is not. Not only does it entrench class‐, gender‐ or race‐based symbolic domination, as others have already suggested, but it also entrenches and diversifies the power of large corporations. In that much it resembles other digital technologies (Savage 2021), and the same may well apply to investment in the many other technologies with immense potential for reshaping human life, from bioengineering and mind‐computer interface to nanotechnology and space exploration. Whatever profound effects these might have on habitus, capitals and fields in the future, in constant need of sociological investigation, we can and should also track the structures and struggles underlying their origins and proliferation so that wherever humanity ends up we know how it all happened, where it all came from and whose interests it served.

## Conflicts of Interest

The author declares no conflicts of interest.

## Supporting information

Supporting Information S1

## Data Availability

The data that support the findings of this study are openly available in UK Data Service at https://beta.ukdataservice.ac.uk/datacatalogue/studies/study?id=8906, reference number 8906.

## Endnotes

^1^
Powell thus brings neo‐institutionalism close to network theories of innovation (e.g. Castilla et al. 2000; Ramella 2019).

^2^
Bourdieu (2005) himself described the network of agents implicated in housing policy as a field, but it never quite fit his core conception of a field. See Atkinson (2020).

^3^
Bourdieu's (2005, 194) clipped definition of financial capital as ‘direct or indirect […] mastery of financial resources’, both ‘actual and potential’, needs specification given the distinction in mainstream economics between financial capital (monetary resources, including investments and retained earnings) and real capital (productive capital, e.g. machines). Bourdieu's definition seems closer to the former, the latter being covered by technological capital, but the latter is also evidently convertible into financial capital through not only productivity but sell‐offs.

^4^
The intellectual field more broadly is also relevant, given the effect of the classic intervention of Hubert Dreyfus (see McCorduck 2004) and the more recent philosophising on AI associated with someone like Nick Bostrom (see McKelvey and Roberge 2023).

^5^
See, for example, the ranking of states produced by the ‘Global Vibrancy Tool’ developed at the Stanford Institute for Human‐Centered AI (https://hai.stanford.edu/news/global‐ai‐power‐rankings‐stanford‐hai‐tool‐ranks‐36‐countries‐ai).

^6^
For details of the study, see the information and report available at the doi: 10.5255/UKDA‐SN‐8906‐1.

^7^
See contributions to, for example, Coulangeon and Duval (2014), Hanquinet and Savage (2018), Korsnes et al. (2018), Hugrée et al. (2023) or the special issue of *European Societies* (2018, vol 20, no. 3) on ‘Toward a European Social Topography’. As far as I am aware, though, this is the first effort to use MCA to model an economic field per se rather than a specific sector (as in Bourdieu 2005, 2008).

^8^
Some low‐n modalities have been set as passive; others have been aggregated where there was propinquity in exploratory models. The FTSE variable is characterised by one large‐n category and two relatively low‐n categories but its *theoretical* importance to the structure of the field warranted inclusion as an active variable.

^9^
Although the modified inertia rate of the second axis is relatively low, its sociological meaningfulness and notable relationships with supplementary variables make it worthy of interpretation.

^10^
The correlation coefficients for technology costs and labour costs on Axis 1 are, respectively, −0.39 (*p* < 0.001) and −0.32 (*p* < 0.001).

^11^
Again, the correlation coefficients for expected 1‐year and 5‐year spend on Axis 1 are −0.31 (*p* < 0.001) and −0.36 (*p* < 0.001), respectively.

^12^
The test values for the modalities on Axis 2 are both notable. Although the SD between them is not notable, a Student's t‐test suggests the difference between the means on the axis to be significant (*t* = 2.8, *p* < 0.01).

^13^
Again, although the SD is not notable, t‐tests suggest the difference between the means for buying software and for developing it in house (*t* = 3.6, *p* < 0.001) or outsourcing it (*t* = 2.4, *p* < 0.05) are significant.

^14^
And to squash small competitors, as with large music labels suing tech startup companies training AI on their artists' catalogues only to develop their own AI technology in the meantime.
