# Supplementary material for: Artificial Intelligence as a Strategy in the British Economic Field
Source: Br J Sociol. 2025 Apr 29;76(4):814–27. doi: 10.1111/1468-4446.13218 (PMC12412078; doi:10.1111/1468-4446.13218)
Supplement: Supplementary file 1 — Supporting Information S1 [file BJOS-76-814-s001.docx]

**Table S1 Coordinates and test values of supplementary variables**

|  |  | Coordinate | | | | Test value | | |
| --- | --- | --- | --- | --- | --- | --- | --- | --- |
|  | n | Axis 1 | | Axis 2 | | Axis 1 | | Axis 2 |
| *Machine Learning* |  |  | |  | |  | |  |
| Not used, no plans | 1119 | 0.42 | | 0.05 | | 21.01 | | 2.35 |
| Not used, plan to | 166 | -0.19 | | -0.04 | | -2.50 | | -0.55 |
| Piloting | 86 | -0.83 | | -0.19 | | -7.90 | | -1.81 |
| Used <3 years | 223 | -0.74 | | -0.14 | | -11.77 | | -2.22 |
| Used 3 years+ | 159 | -0.88 | | -0.07 | | -11.59 | | -0.88 |
| Missing | 256 | -0.23 | | 0.05 | | -3.97 | | 0.86 |
|  |  |  | |  | |  | |  |
| *Language modelling* |  |  | |  | |  | |  |
| Not used, no plans | 1125 | 0.38 | | 0.07 | | 19.29 | | 3.70 |
| Not used, plan to | 167 | -0.05 | | 0.06 | | -0.66 | | 0.75 |
| Piloting | 103 | -0.69 | | -0.19 | | -7.20 | | -2.01 |
| Used <3 years | 205 | -0.78 | | -0.21 | | -11.85 | | -3.17 |
| Used 3 years+ | 154 | -0.81 | | -0.18 | | -10.40 | | -2.30 |
| Missing | 255 | -0.26 | | -0.01 | | -4.36 | | -0.09 |
|  |  |  | |  | |  | |  |
| *Computer Visualisation* |  |  | |  | |  | |  |
| Not used, no plans | 1255 | 0.35 | | 0.02 | | 20.06 | | 1.07 |
| Not used, plan to | 112 | -0.27 | | -0.02 | | -2.89 | | -0.19 |
| Piloting | 78 | -0.75 | | -0.26 | | -6.74 | | -2.38 |
| Used <3 years | 180 | -0.82 | | -0.07 | | -11.46 | | -0.93 |
| Used 3 years+ | 132 | -0.78 | | 0.21 | | -9.24 | | 2.47 |
| Missing | 252 | -0.39 | | -0.06 | | -6.59 | | -1.09 |
|  |  |  | |  | |  | |  |
| *Data Management* |  |  | |  | |  | |  |
| Not used, no plans | 957 | 0.49 | | 0.05 | | 20.88 | | 1.93 |
| Not used, plan to | 200 | -0.12 | | -0.07 | | -1.78 | | -1.00 |
| Piloting | 92 | -0.77 | | -0.08 | | -7.55 | | -0.79 |
| Used <3 years | 255 | -0.62 | | -0.05 | | -10.55 | | -0.86 |
| Used 3 years+ | 241 | -0.73 | | -0.07 | | -12.08 | | -1.24 |
| Missing | 264 | -0.15 | | 0.03 | | -2.62 | | 0.57 |
|  |  |  | |  | |  | |  |
| *Hardware* |  |  | |  | |  | |  |
| Not used, no plans | 1284 | 0.32 | | -0.01 | | 19.31 | | -0.47 |
| Not used, plan to | 124 | -0.19 | | -0.01 | | -2.17 | | -0.13 |
| Piloting | 83 | -0.96 | | -0.06 | | -8.93 | | -0.58 |
| Used <3 years | 137 | -0.80 | | -0.07 | | -9.72 | | -0.86 |
| Used 3 years+ | 131 | -0.81 | | 0.15 | | -9.55 | | 1.82 |
| Missing | 250 | -0.39 | | 0.02 | | -6.56 | | 0.42 |
|  |  |  | |  | |  | |  |
| *Robotics* |  |  | |  | |  | |  |
| Not used, no plans | 1158 | 0.40 | | 0.02 | | 21.07 | | 1.04 |
| Not used, plan to | 125 | -0.18 | | -0.03 | | -2.08 | | -0.34 |
| Piloting | 71 | -0.87 | | -0.23 | | -7.50 | | -1.96 |
| Used <3 years | 204 | -0.83 | | -0.04 | | -12.48 | | -0.57 |
| Used 3 years+ | 217 | -0.67 | | 0.07 | | -10.49 | | 1.17 |
| Missing | 234 | -0.29 | | -0.05 | | -4.66 | | -0.80 |
|  |  |  | |  | |  | |  |
| *Mode of adoption* |  |  | |  | |  | |  |
| Developed in-house | 267 | -0.73 | | -0.12 | | -12.84 | | -2.10 |
| Outsourced | 146 | -0.65 | | 0.18 | | -8.10 | | 2.26 |
| Purchased software | 287 | -0.41 | | -0.05 | | -7.53 | | -0.91 |
| Other | 9 | 0.04 | | -0.11 | | 0.11 | | -0.32 |
| Missing | 1300 | 0.31 | | 0.02 | | 19.02 | | 0.98 |
|  |  |  | |  | |  | |  |
| *Increase in spend next year* | |  | |  | |  | |  |
| <50% | 474 | -0.44 | | -0.02 | | -11.01 | | -0.57 |
| >50% | 93 | -0.94 | | -0.16 | | -9.30 | | -1.54 |
| Missing | 1442 | 0.21 | | 0.02 | | 14.73 | | 1.26 |
|  |  |  | |  | |  | |  |
| *Increase in spend next 5 years* | |  | |  | |  | |  |
| <50% | 382 | -0.34 | | -0.04 | | -7.42 | | -0.96 |
| >50% | 182 | -0.93 | | -0.07 | | -13.11 | | -0.94 |
| Missing | 1445 | 0.21 | | 0.02 | | 14.85 | | 1.43 |
|  |  |  | |  | |  | |  |
| *Costs on AI tech last 3 years* | | |  | |  | |  | |
| <£1m | 412 | -0.35 | | -0.03 | | -7.96 | | -0.63 |
| >£1m | 109 | -1.29 | | -0.10 | | -13.90 | | -1.07 |
| Missing | 1488 | 0.19 | | 0.01 | | 14.52 | | 1.14 |
|  |  |  | |  | |  | |  |
| *Costs on AI labour last 3 years* | | |  | |  | |  | |
| <£1m | 385 | -0.40 | | -0.01 | | -8.67 | | -0.19 |
| >£1m | 99 | -1.33 | | -0.17 | | -13.52 | | -1.77 |
| Missing | 1525 | 0.19 | | 0.01 | | 14.82 | | 1.07 |
| *Note:* test values greater than +/-2 are notable. | | | | | | | | |

**The internal dispersion of ICT, infrastructure and construction firms**

The middling positions of certain industry categories on Axis 1 of the space reflect their internal dispersion along the axis (Figure S1). Hence, whereas businesses in retail, education (etc), arts/leisure and other sectors predominantly fall into the bottom tercile of the space, and while *no* finance enterprises fall into the bottom tercile, the manufacturing, infrastructure and ICT sectors are characterised by a fairly even split between higher- and middle-placed firms plus a rump of businesses characterised by low turnover, small size, and regional location. In the case of ICT, this rump may denote small-scale ‘tech start ups’ or specialised business-to-business operations and consultancies. Construction differs inasmuch as a larger proportion falls into the bottom tercile of the space and a smaller proportion sit within the top tercile. The largest percentage of construction firms (46 percent) falls into the middle section. If any specific industry were to be characterised as typically ‘petit bourgeois’, construction would be the most likely candidate.

**Figure S1 The proportion of businesses falling into the lower, middle and top terciles on Axis 1 by sector.**
